# Comparing microfluidics and ultrasonication as formulation methods for developing hempseed oil nanoemulsions for oral delivery applications

**DOI:** 10.1038/s41598-020-79161-w

**Published:** 2021-01-08

**Authors:** Farahnaz Fathordoobady, Natalia Sannikova, Yigong Guo, Anika Singh, David D. Kitts, Anubhav Pratap-Singh

**Affiliations:** 1 Food Nutrition and Health Program, Faculty of Land and Food Systems, The University of British Columbia, 2205 East Mall, Vancouver, BC V6T 1Z4 Canada; 2 Ascension Sciences Inc, Vancouver, BC Canada

**Keywords:** Nanoscale biophysics, Design, synthesis and processing, Biomedical engineering, Chemical engineering, Drug delivery

## Abstract

Emerging formulation technologies aimed to produce nanoemulsions with improved characteristics, such as stability are attractive endeavors; however, comparisons between competing technologies are lacking. In this study, two formulation techniques that employed ultrasound and microfluidic approaches, respectively, were examined for relative capacity to produce serviceable oil in water nanoemulsions, based on hempseed oil (HSO). The ultrasound method reached > 99.5% entrapment efficiency with nanoemulsions that had an average droplet size (Z-Ave) < 180 nm and polydispersity index (PDI) of 0.15 ± 0.04. Surfactant concentration (% w/v) was found to be a significant factor (*p* < 0.05) controlling the Z-Ave, PDI and zeta potential of these nanoparticles. On the other hand, the microfluidic approach produced smaller particles compared to ultrasonication, with good stability observed during storage at room temperature. The Z-Ave of < 62.0 nm was achieved for microfluidic nanoemulsions by adjusting the aqueous : organic flow rate ratio and total flow rate at 4:1 and 12 mL/min, respectively. Further analyses including a morphology examination, a simulated gastrointestinal release behavior study, transepithelial transport evaluations and a toxicity test, using a Caco2-cell model, were performed to assess the functionality of the prepared formulations. The results of this study conclude that both approaches of ultrasound and microfluidics have the capability to prepare an HSO-nanoemulsion formulation, with acceptable characteristics and stability for oral delivery applications.

## Introduction

The development of nanoemulsions (NEs) for use in the delivery of pharmaceuticals or nutraceuticals, that otherwise have poor aqueous solubility, has resulted in a number of successful lipid-carrier formulations. NEs can exist as both an oil-in-water (O/W) and water-in-oil (W/O) emulsion, where the dispersed phase contains a moderate concentration of emulsifying agent (4–8 wt%). NEs present in a heterogeneous phase are nanoscale (50–500 nm) size droplets that coexist in a continuous phase stabilized by emulsifiers or surfactants^1^. The typical structure of emulsifiers are non-polar hydrophobic tails and polar head groups that enable effective absorption at the interface of an oil and water emulsion by decreasing interfacial tension between the two immiscible phases^2^. Using NEs, for the oral delivery of drugs, should minimize the required amount of emulsifier without losing internal phase components^3^. An ideal amount of emulsifying agent, whether it be non-ionic surfactant like lecithin, poloxamers, or non-electrolytes such as glycerol or xylitol, is an important consideration in developing an optimal NE formulation^4^. Generally, the hydrophilic-lipophilic balance (HLB) of the emulsifier will assist to identify the best suited stabilizer; for example a low HLB number (e.g. 3–6) favors a W/O emulsion, while a high HLB (e.g. 8–13) are used to stabilize O/W emulsion systems^5^. In some cases, the HLB number decreases with increasing temperature; thus, a phase inversion temperature (PIT) is required to decide on the emulsifier best suited to stabilize emulsions. Recent technological advances that enhance ease of production, high stability and capacity to solubilize hydrophobic agents mark new developments for using NEs for many applications^2^.

NEs that achieve a nanoscale size dispersed droplet require high stability to be effective. Applying high shear forces to the liquid phase will produce this effect. High-energy techniques are often used to prepare NEs including ultrasonication, microfluidization and high-pressure homogenization. Ultrasonication efficiently reduces particle size in small scale production systems^6^. The energy introduced by soundwaves results in cavities and sinusoidal pressure changes at a liquid–liquid interphase that translates to a shock-wave effect on the particle surface resulting in reduced particle size. The ultimate size of particles depends on frequency, amplitude and the sonication process^4^. Microfluidics have been described as systems that process or manipulate micro- or nano-liter volumes of liquids through microchannels, with transport controlled by mixing, heat, mass transport and particle properties^7,8^. This technology offers inherent properties such as producing high surface area to volume ratio to test micro/nano particles for drug delivery. Preparing nanoparticles with microfluidic technology to encapsulate bioactive agents also involves using devices that manipulate liquids in microchannels with high ratios of surface area to volume, and efficient mass and heat transfer^9,10^.High processing accuracy and efficiency, the ability to establish a multistep platform design, cost savings and reduced consumption of hazardous reagents are some of the additional advantages that this technology enables^11^.

In our previous work, we demonstrated how selective antioxidants regulate the rate of fatty acid degradation and decarboxylation of acidic cannabinoids which exist in hempseed oil^12^. Based on these previous results, we utilized an ultrasound process and microfluidic technology in this study to produce O/W NEs with hempseed oil (HSO) as the dispersed phase. Hempseed oil derived from *Cannabis sativa L*. has been shown to have nutritional value due to a notable content and ratio of omega-6 and omega-3 fatty acids, as well as the presence of γ-linolenic acid, stearidonic acid, tocopherols, tocotrienols, carotenes, minerals, β-sitosterols and terpenoids that collectively contribute to oxidative stability^12,13^. Moreover, of particular interest is the presence of non-psychoactive cannabinoid compounds, that include cannabidiolic acid (CBDA) and cannabidiol (CBD) found in HSO^14^. These compounds are biologically active and demonstrate anti-convulsive, anti-epileptic and antimicrobial effects^15^. HSO based NEs are potential delivery vehicles that could be utilized as a carrier of other therapeutic agents with poor water solubility. The purpose of the current study was to investigate the practicality of employing microfluidic or ultrasound technology to develop HSO NEs. We also assessed the stability, shelf-life storage capacity and possible effects of bioactives present in HSO based NEs; the later employing cellular bioavailability assessments to determine practical applications.

## Materials and methods

### Materials

Unrefined cold-pressed hempseed oil (HSO) was purchased from Manitoba Harvest Hemp Foods (Winnipeg, Manitoba, Canada). Refined lecithin (MW = 758.075 g/mol, Purity > 98.0%) was obtained from Alfa Aesar Co. Inc (Mississauga, Ontario, Canada). Poloxamer 188 (MW ~ 8400 g/mol) was purchased from Corning Co. Inc (Manassas, Virginia, USA). FAME (fatty acid methyl ester) mix containing 14 components provided by Restek Co. (Bellefonte, PA, USA) was used for fatty acid analysis. All other reagents and chemicals were purchased from Sigma-Aldrich Canada Corp (Ontario, Canada) and were analytical or chromatography grade.

### Fatty acids analysis of HSO and nanoemulsions

#### Sample preparation for GC/MS analysis

The fatty acid profile of samples was determined according to procedures reported earlier^14^. Fatty acid methyl esters (FAMES) were analyzed by GC/MS using a PerkinElmer model operated with Clarus 680-GC-SQ8T Mass Spectrometer (MA. USA). Helium (99.99%) was used as carrier gas with a flow rate of 1 mL/min. A volume of 1 μL prepared sample was injected at 250 °C in a split mode (50:1). The temperature was programmed according to: 150 °C initial temperature held for 2 min; increased to 185 °C at a rate of 1.5 °C/min; reached a final temperature of 220 °C at a rate of 5.0 °C/min. MS conditions were 70 eV ionization energy with 250 °C ion source temperature. The mass to charge (*m/z*) range was set as 20–450 atomic mass units. TurboMass Ver. 2.3 (NIST 2011, Waltham, MA, USA) software was used to determine the fatty acids profile of HSO and nanoemulsions.

#### NMR studies for HSO

We used NMR analysis to confirm the polyunsaturated and saturated fatty acid content (PUFA and SFA) of HSO. Samples for analysis were prepared as follows: HSO (200 μL) was dissolved in 800 μL of CDCl_3_. ^1^H NMR spectra were recorded on a Bruker Avance Cryoprobe 600 MHz spectrometer (MA, USA). Chemical shifts are reported in parts per million downfield from TMS. Spectra were processed using MestReNova Ver. 12.0.4 (Mestrelab Research, Santiago de Compostela, Spain).

### Cannabinoids analysis with LC–MS system

LC–MS analyses were carried out according to Citti et al. (2018) to identify the cannabinoid content of HSO^16^. An Agilent 1290 Infinity/6530 Accurate Mass Q-TOF system equipped with MassHunter Workstation (B.07.00) software was used for this analysis. The Agilent Zorbax Eclipse Plus column (C18, 2.1 × 50 mm) with 1.8 μm pore size was used. The mass spectrometer was operated in both ESI + and ESI− ionization mode at a dry gas flow rate of 12 mL/min, the nebulizer (N_2_) pressure of 60 psi and 400 °C temperature; 65 V skimmer voltage; and 4.0 kV set capillary voltage. The mass range of 50–1000 m/*z* was used for 2.00 μL of injected sample. Extracted ion chromatograms (EICs) were obtained by applying the *m*/*z* corresponding to the molecular ions [M + H].

### Nanoemulsion preparation

#### Ultrasound technique for preparing hempseed oil nanoemulsion

A two-step process, described earlier^17^ with some modifcations, was used to prepare the O/W nanoemulsions. The pre-emulsions were prepared using a 1200 W Polytron PCU-2–110 high speed homogenizer (Brinkmann Instruments, Inc., Westbury, NY, USA). The hempseed oil was mixed in with distilled water to obtain a 50 mL solution that also contained lecithin (emulsifier) and poloxamer 188 (co-emulsifier) in concentrations described in supplement Table 1. The mixture was then homogenized for 10 min at 10,000 rpm. These pre-emulsions were processed further by an ultrasonicator (Hielscher Ultrasonics, Teltow, Germany) for a specified time outlined in experimental design (see supplement Table 1) using 40% full power (20 kHz frequency) in continuous mode to obtain the final emulsion. An ice-bath was used to control the temperature during the ultrasonication process. All nanoemulsion samples were stored at 4 °C for further analyses.

#### Microfluidic technology for preparing hempseed oil nanoemulsion

**Figure 1:**
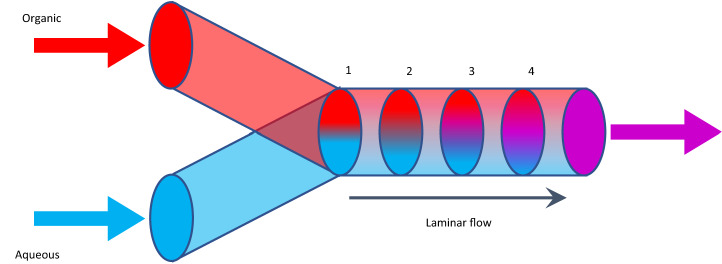
Controlled solvent displacement in a microfluidic mixer: (1) Organic (ethanol) and aqueous (PBS) phases enter the channel under laminar flow without mixing; (2) Microscopic features in the channel cause fluid streams to mix in a controlled fashion; (3) Intermingling of the phases increases through the mixer; (4) Fluids emerge, mixed as a stable emulsion.

A NanoAssemblr Benchtop instrument (Precision NanoSystems) was used to prepare the O/W nanoemulsions. The disperse, organic phase was comprised of an ethanolic solution containing surfactants (Tween 80: Span 80; 2.3:1 v/v) with different concentrations of hempseed oil. Deionized water was used as a continuous aqueous phase. Instrument parameters total flow rate and flow rate ratio (aqueous: organic) were varied in the ranges of 2–12 mL/min and 1:1–4:1, respectively, in order to determine their effect on emulsion droplet size and stability. Ethanol was removed by dialysis (10 kDa MWCO dialysis bags) in deionized water at room temperature over 24 h and then samples were stored at 4 °C post dialysis. The displacement of organic and aqueous phases in a microfluidic mixer is shown schematically in Fig. 1.

### Experimental design and statistical analysis

A response surface methodology (RSM) design (Minitab software version 18.0, Minitab LLC, State College, PA, USA) was used for the optimization of nanoemulsion preparation. The variables included HSO content (% v/v), lecithin content (% w/v), poloxamer content (% v/v), and ultrasound processing time (min) at five coded levels (− *α*, − 1, 0, + 1, + *α*). The central composite design (CCD) provided 30 experimental trials including 16 trials for the factorial design, 8 trials for axial and 6 trials for central point replications. The responses were fitted to a non-linear model, as shown in Eq. (1).

1 $$\mathrm{Y }= \beta _{0} + \beta _{1}x_{1} + \beta _{2}x_{2} + \beta _{3}x_{3}+ \beta _{4}x_{4} + \beta _{1sup2}(x_{1})^{2} + \beta _{2sup2}(x_{2})^{2} +\beta _{3sup2}(x_{3})^{2} +\beta _{4sup2}(x_{4})^{2} + \beta _{1-2}x_{1}x_{2} + \beta _{1-3}x_{1}x_{3} + \beta _{1-4}x_{1}x_{4} + \beta _{2-3}x_{2}x_{3} + \beta _{2-4}x_{2}x_{4} + \beta _{3-4}x_{3}x_{4}$$

where Y is the response variable; *β*_*0*_ the constant; *β*_*1*_–*β*_*4*_ were linear coefficients; *β*_*1-2*_–*β*_*3-4*_ were the interaction effect coefficients; *β*_*1sup2*_–*β*_*4sup2*_ were the coefficients for the quadratic effect and *x*_*1-4*_, represent the independent variables. Low and high factor settings as well as the central point were coded as − 1, + 1, and 0, respectively. The experiments were randomized to reduce the unexplained effects of response variability. Nanoemulsion preparation conditions were optimized numerically and graphically to obtain the desired values for response variables that included particle size (nm), polydispersity index (PDI) and Zeta potential.

### Droplet size analysis

Hydrodynamic diameters of emulsion droplets were determined using dynamic light scattering (DLS) technique (Litesizer 500; Anton Paar, Graz, Austria). Measurements were performed using automatic settings adjusted for the latex spheres at 25 °C. The concentration of samples was 1 mg/mL. All measurements were performed in triplicate, with mean values determined. The Zeta potential (ζ) (mV) of particles was assayed using an Omega cuvette Z (Anton Paar, Graz, Austria). The morphology of the nanoemulsions was examined by transmission electron microscopy (TEM) (Hitachi H7600, Tokyo, Japan). The resulting images were analyzed using Hitachi imaging software (Hitachi, Tokyo, Japan).

### Entrapment efficiency (%)

Linoleic acid (LA) was used as the chemical marker to determine the entrapment efficiency of HSO within the emulsion structure. Samples were first centrifuged at 12,000 rpm at 15 °C using a Legend X1R centrifuge system (Thermo Fisher Scientific, Inc., Waltham, MA, USA) for 15 min to separate nanoemulsion particles from the unbound oil in the aqueous phase. The supernatant was collected and fatty acids, including LA, were measured using a GC-FID. LA entrapment efficiency (%) was determined according to Eq. (2).

2 $$Entrapment\;efficiency\left( \% \right) = \left( {{\raise0.7ex\hbox{${1 - Determined\;LA}$} \!\mathord{\left/ {\vphantom {{1 - Determined\;LA} {Total\;LA}}}\right.\kern-\nulldelimiterspace} \!\lower0.7ex\hbox{${Total\;LA}$}}} \right) \times 100$$

### In-vitro release characteristics

HSO released from nanoemulsions prepared using the ultrasound technique was recovered by dialysis. Nanoemulsions with different HSO content were dialyzed in a simulated gastrointestinal fluid that was composed of a HCl solution (pH 1.3) and phosphate buffered saline (PBS), pH 7.0. All samples were incubated at 37 °C with continuous shaking. Samples (5 mL) were withdrawn at the following times: 0.2, 0.4, 1, 2, 3, 4, 6, 8, and 12 h, and the volume immediately replenished with fresh dialysis fluid. Samples were analyzed for fatty acids by GC-FID and the release rate of HSO from nanoemulsion was calculated from the ratio of released as free LA to the total LA entrapped in nanoemulsion system (Eq. 3).

3 $$Release rate = Released free LA/ total LA$$

### Transepithelial transport property of NEs

**Figure 2:**
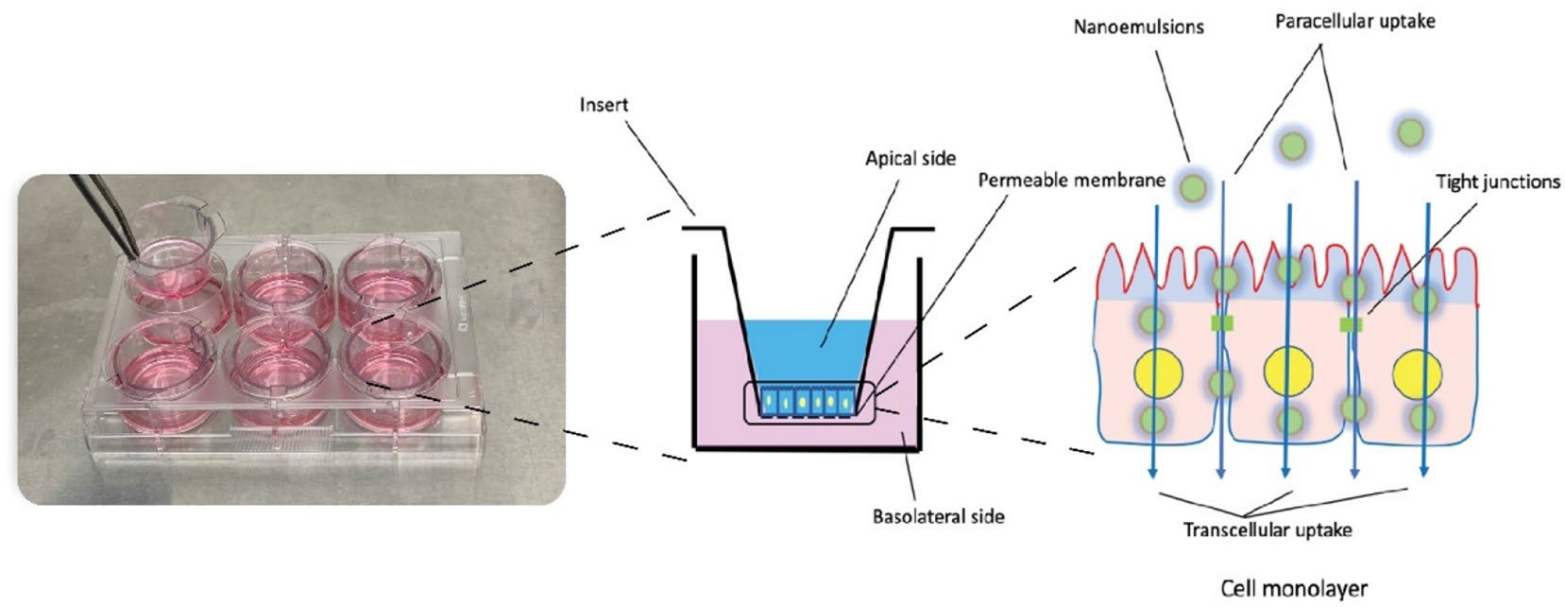
Transepithelial transport study of nanoemulsions using Caco-2 cells model.

Differentiated Caco-2 cells were used to determine the trans-epithelial property of nanoemulsions. Cells were maintained in Dulbecco’s modified Eagle’s medium (DMEM) containing 1% penicillin–streptomycin and 10% fetal bovine serum (FBS) in a humidified standard incubator, set at 37 °C, 5% CO_2_. Caco-2 cells were seeded in six-well plates under cover glass at a density of 1 × 10^5^ cell per well and cultured for 21 days to establish differentiation before treatment. The formation of the monolayers was confirmed before and after the experiment by transepithelial electric resistance (TEER) value using the Millicell-ERS-2system (Millipore Corporation, Bedford, MA). Prior to conducting experiments, media in the apical and basolateral chambers were washed with phosphate buffered saline (PBS) three times and allowed to equilibrate at 37 °C for 30 min. Nanoemulsions (200 μL) and HSO diluted in PBS 12.5% (w/v) were added into the apical chamber and incubated for 24 h. At determined time points (1, 2, 4, 12 and 24 h after incubation), the medium from the basolateral chamber was sampled to determine LA transported, using GC-FID analysis. Figure 2 shows a scheme of the Caco-2 cells test used for the nanoemulsion transport study.

### Toxicity studies

Assessment of potential cytotoxicity of prepared nanoemulsions was performed using a Caco-2 cell viability test, or MTT (3-(4, 5-dimethylthiazol-2-yl)-2,5-diphenyltetrazolium bromide) assay. Caco-2 cells were seeded in 96 well plates at a density of 1 × 10^5^ cells/well and cultured for 21 days. The cells were incubated with 100 μL of nanoemulsion at 125 mg/mL HSO for 24 h at 37 °C, before adding MTT. Changes in cell redox, an indicator of viability, was recorded at 550 nm wavelength. Tests were performed in triplicate, and data were expressed as the percentage of viable cells exposed to nanoemulsion compared to control.

## Results and discussion

Prior to preparing the nanoemulsions, some of the important nutritional characteristics of HSO including fatty acids profile and cannabinoids composition of the hempseed oils were determined.

### Fatty acids profile of HSO used for nanoemulsions

The fatty acid content of HSO used for preparing nanoemulsion samples is represented in Supplement Table 1. Palmitic (C16:0), oleic (C18:1), linoleic (C18:2), and α-linolenic (C18:3), acids are the principle unsaturated and saturated fatty acids, respectively present in HSO; which also contained γ -linolenic acid. Previous investigations from our laboratory reported similar proportions of monounsaturated LA in HSO, but in lower quantities^13^. In our present study, we also detected small quantities of stearidonic and eicosenoic acids, also reported by others^14^. The degree of unsaturation (91.0%) and the ratio of 3.25/1 for ω-6/ω-3 fatty acids remained in agreement with what we have reported earlier^12^ in accordance with Da Porto et al., 2012, Teh, & Brich, (2013), and Gao & Birch (2016)^18–20^.

Using ^1^H NMR, we confirmed the HSO fatty acid compositions formerly reported^21^. LA (ω-6)/ α-linolenic acid (ω-3), and LA (ω-6)/oleic acid (ω-9) ratios were 3.0 and 2.16, respectively. The ratio of total saturated to unsaturated acid was 0.11 (See Supplement Fig. 1).

### Cannabinoids content of HSO

In this study, the mass-to-charge (M/Z) LC–MS chromatograms for cannabinoid compounds including non-psychoactive cannabidivarin (CBDV), cannabidiolic acid (CBDA), cannabidiol (CBD), cannabigerol (CBG), and small quantities of psychoactive tetrahydrocannabivarin (THCV), tetrahydrocannabinolic acid (THCA), and tetrahydrocannabinol (THC) were identified (See Supplement Fig. 2) as we also reported in our previous work^12^. We expected that THC in HSO is detected in only a trace amount due to contamination of HSO during the oil seed pressing process^16^. Indeed, we found CBDA to be the principle cannabinoid and THC to be present at a negligible amount (< 0.2%) in HSO. Citti et al. reported higher concentrations of CBDA, CBD and CBN, compared to THCA and THC, in HSO processed from hulled seeds. CBN was not detected in our HSO sample which contrasts former studies^16,22^.

### HSO nanoemulsion process optimization, characterizations, and stability

HSO, O/W nanoemulsions were prepared using two different procedures and formulations. These involved utilizing an ultrasound processor and microfluidic technology, as well as using various types and concentrations of emulsifiers to ensure an HLB value between 6 and 10, thus characterizing an O/W emulsion. Key characteristics of a constructed nanoemulsion are changes that affect emulsion stability, such as particle size and distribution of the particles. In this study, the methods used to prepare stable nano-emulsion system was investigated separately.

#### Ultrasound process optimization

The O/W nanoemulsions were prepared based on an RSM-CCD experimental design with four independent variables that included HSO (5–10%), poloxamer (1–5%), lecithin (0–5%) and a process mixing time (5–20 min) (See supplement Table 1). The ranges were based on preliminary studies performed by authors. In this study, four response variables of particle size (nm), PDI, zeta potential and EE (%) were re-tested to evaluate the stability and potential of prepared emulsion for its usage as a lipid carrier.

The reduced models for each variable are described by Eqs. (4–7):

4 $${y}_{1}=351-21.9{x}_{1}+0.8{x}_{3}+4.03{x}_{3}^{2}+0.437{x}_{4}^{2}+5.88{x}_{1}{x}_{2}$$

5 $${y}_{2}=0.769-0.050{x}_{3}+0.009{x}_{3}^{2}$$

6 $${y}_{3}=46.1+3.49{x}_{3}-0.631{x}_{3}^{2}+0.412{x}_{2}{x}_{4}$$

7 $${y}_{4}=29.0+13.57{x}_{3}$$

where *y*_1_, *y*_2_, *y*_3_ and *y*_4_ are response variables of particle size (nm), PDI, Zeta potential and EE (%), respectively, and the variables, o*x*_1_, *x*_2_, *x*_3_ and *x*_4_ represent HSO (%); poloxamer (%); lecithin (%), and time used for ultrasound mixing.

From these equations, it was concluded that the lecithin concentration was a major factor that influenced all response variables significantly (*p* < 0.05) and was a critical parameter to govern the PDI and EE (%) of the emulsion system (Eqs. 5 and 7). The concentration of HSO independently and in combination with poloxamer concentration affected the particle size of nanoemulsion. In this experiment, the time of mixing was described by a quadratic equation with significant variable interactions (*p* < 0.05) that affected particle size and zeta potential of nanoemulsion droplets.

The relative significance of individual variables was ranked according to the greatest effect in relative order of lecithin (%) > HSO (%) > process mixing time (min) > poloxamer (%), to explain emulsion stability. Generally, emulsifiers with HLB values between 8 and 13 are preferable to prepare O/W emulsion^5^. Emulsions containing soybean lecithin (HLB value ranging between 4–8) versus poloxamer (HLB value of 29) were needed to contain optimal proportions to obtain the desired effect. For example, although poloxamer concentration in this experiment had no significant effect (*p* < 0.05) on response variables, its presence in the formulation was required to produce a stable HSO nanoemulsion. It was also noticed that with using HSO concentrations greater than 8%, increasing the poloxamer concentration resulted in larger particle sizes. This outcome indicated that the interaction of factors (*x*_1_*x*_2_) was not in favor of reducing particle size. These observations implied that the ratio of HSO to the amount of emulsifier/co-emulsifier require optimization in order to obtain a more stable nanoemulsion system with reduced particle size. The process mixing time, using ultrasound, had a significant quadratic effect (*p* < 0.05) on particle size which indicated that reducing particle size behavior was predictable in our mathematical model.

**Figure 3:**
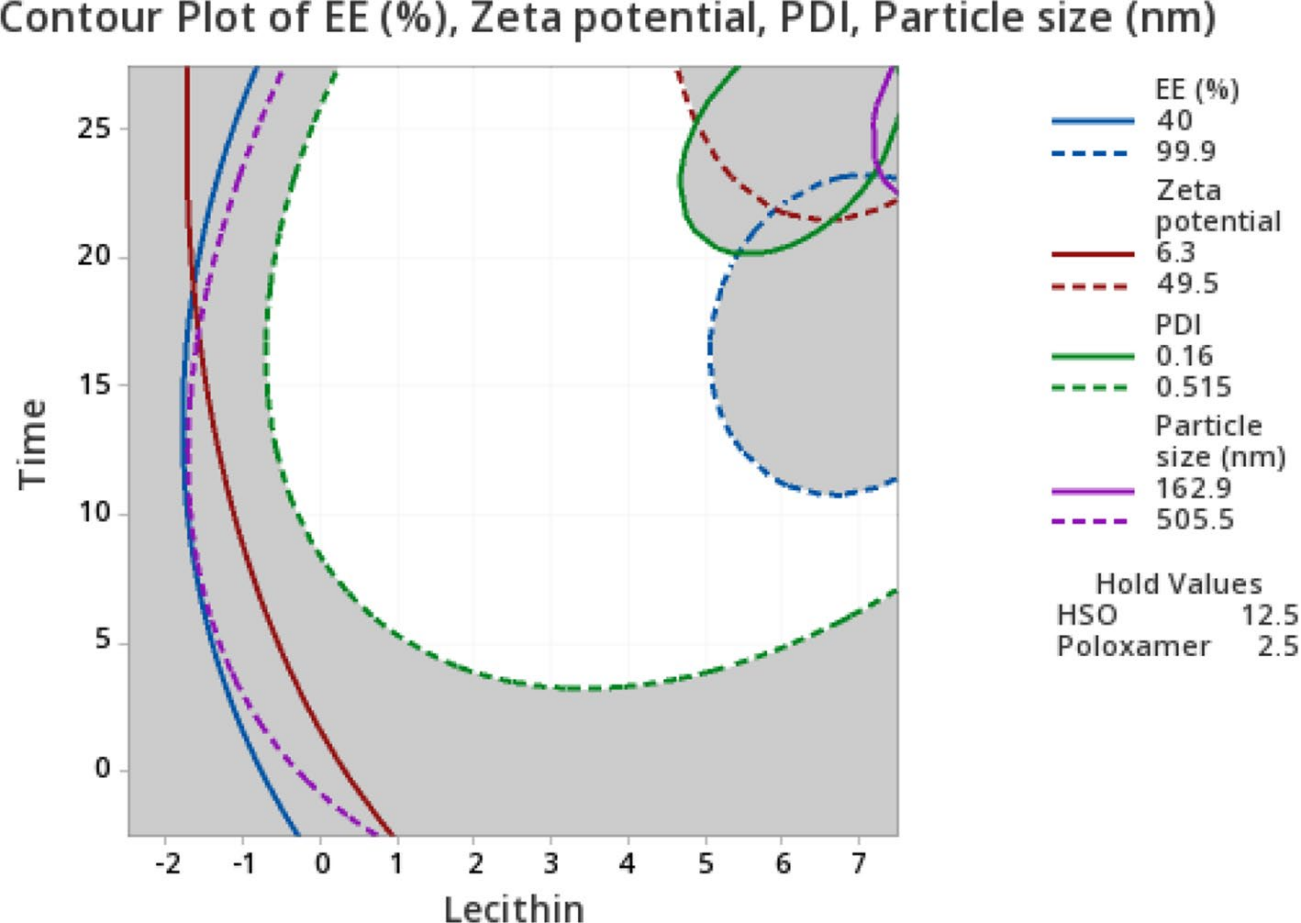
Contour plot of process time (min) and Lecithin (%) for entrapment efficiency (%), Zeta potential (ζ) mV, PDI and particle size (nm).

The contour plot of lecithin concentration and process mixing time were the most significant factors (*p* < 0.05) affecting response variables, that included having 12.5% (w/v) HSO and 2.5% (w/v) poloxamer; and applying a mixing process time of 22 min. Moreover, the lecithin content of this formulation was kept at 7% (w/v) to achieve smaller particle size and PDI values (Fig. 3). Relying on numerical and graphical optimization methods, we showed that optimal nanoemulsion, particle size of 179.4 nm, PDI of 0.15, zeta potential (ζ) of 49.5 (mV) with 99.8 EE (%), was predicted to be achieved using 12.5% (v/v) HSO, 2.47% (v/v) poloxamer 188, 5.98% (v/v) lecithin and 22.4 (min) ultrasound process with the composite desirability of 0.987 (See supplement Fig. 3). This optimal (*p* < 0.05) condition was verified and shown to be highly suitable for a stable nanoemulsion system.

**Figure 4:**
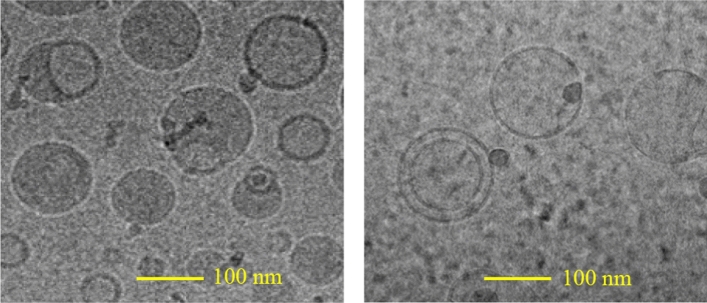
Cryo-electron microscopy of nanoemulsion formulation prepared by hempseed oil + lecithin + poloxamer 188 (30 mg/mL concentration).

A morphology examination of the nanoemulsions developed using optimal conditions described herein, was performed using TEM; a practical technique that captures simultaneous structures and microstructure transitions with high resolution images^23^. The TEM images showed the spherical appearance of droplets with uniform size (Fig. 4). These results were correlated with the average droplet size determined by the particle size analyzer system (~ 180 nm). These nanoemulsions were found to be stable for upto 4 months at 22 °C and 4 °C storage.

### Effect of microfluidic process condition and formulation on droplet size, and storage stability of HSO nanoemulsion

The most important parameters affecting microfluidics are often viscosity, flow rate, and choice of surfactant. Selection of a proper immiscible phase as well as the geometry of the channel are also important in segmented flow system^11,24^. In this study, instrumental configuration including total flow rate (TFR) and aqueous: organic flow rate ratio (FRR) were set at 2–12 mL/min and 1–4:1 for selected formulations that included HSO and surfactants. Emulsion droplet size and PDI were investigated, with stability of the prepared emulsion held for 35 days at 4 °C.

**Figure 5:**
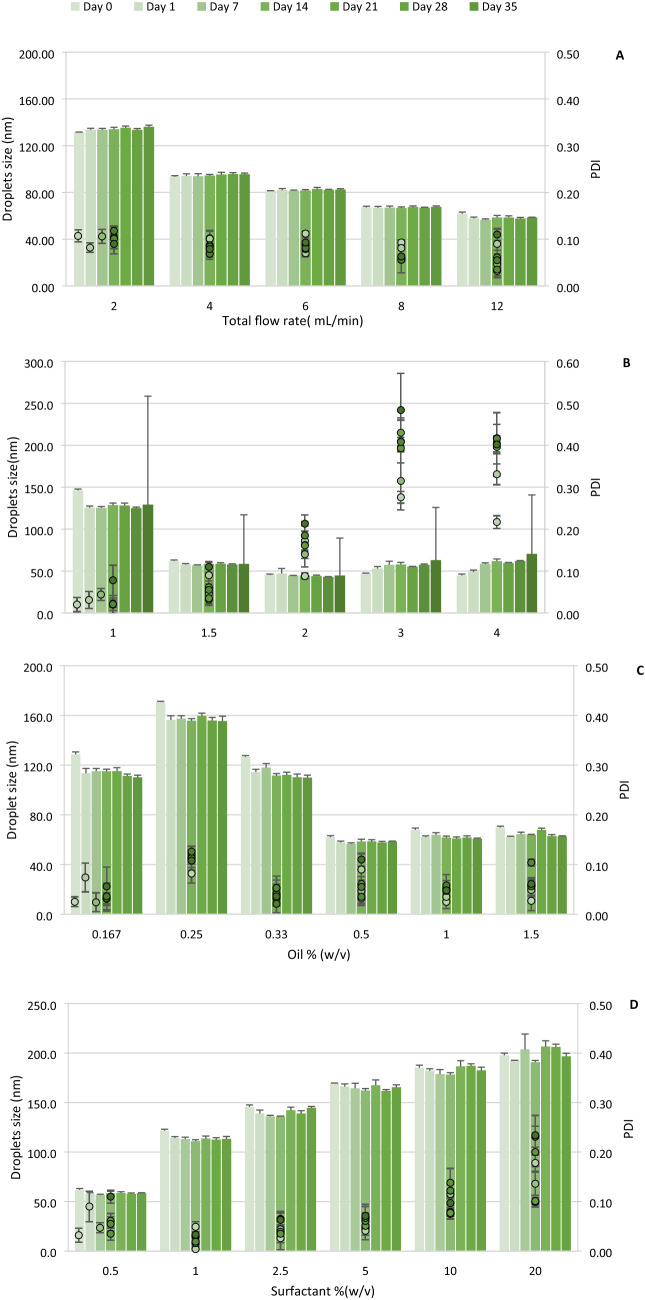
Effect of formulation parameters on mean droplet size (bars) and PDI (points) of HSO nanoemulsion prepared by microfluidic mixing upon 35 days of storage at different: (**A**) Total flow rate (TFR); (**B**) Flow rate ratio (FRR); (**C**) Oil concentration; (**D**) Surfactant concentration. Each point represents the mean value ± SD (*n* = 3).

The effect of varying flow rates on the emulsion average particle size and PDI is shown in Fig. 5A. All samples had a concentration of 0.5% (w/v) HSO, and the total flow rate (TFR) increased from 2–12 mL/min. Under these conditions, the total flow rate was shown to be inversely proportional to the emulsion droplet size when all other parameters were kept constant. Applying a total flow rate of ≥ 4 mL/min produced droplets with average sizes that ranged from 61.95 ± 1.31 to 93.40 ± 1.00 nm over 35 days of storage. The PDI of emulsions prepared using a 2–10 mL/min total flow rate showed no difference during storage. Samples tested at 12 mL/min flow rate had an average PDI of 0.032 ± 0.014, with the emulsion stability being lower due to increased values found for PDI during storage time. The same trend was observed in a previous study when preparing PLGA nanoparticles in two aqueous phases containing sodium dodecyl sulfate and polyvinyl alcohol as emulsifying agents. While the continuous flow rate was increased by 57.1%, the average particle size was reduced by 92.0%^25^. The increase in the TFR would lead to reduced droplet size due to a faster process of droplets forming, cutting at the outlet region. This observation was common for both O/W and W/O emulsions. Gu et al. observed a decrease in droplet size for a W/O dodecane emulsion with increasing total flow rate^26^. Similar findings regarding the relationship between continuous flow rate and droplet size of nanoemulsion have been previously reported^27,28^. Eventually, the stability of the emulsions was found to be similar to as reported above for a period of 4 months of storage at 22 °C and 4 °C. Thus, it was demonstrated that the microfluidic-based platform has the capability to reduce possible shortcomings associated with processing techniques. In addition to decreasing the quantity of required reagents and samples for assessment of functionality, the main advantages of using microfluidic approach were to control precisely the droplet size by adjusting the flow rates of fluids and to formulate in a controlled setting using small volumes of fluids with reduced time for preparing the nanoemulsion.

In our study we found that emulsion droplet size was inversely proportional to organic flow rate ratios (FRRs) when other parameters including total flow rate, and surfactant: oil ratios were held constant. Applying ≥ 1.5 FRRs led to a significant reduction (p < 0.05) of nanoemulsion particle size with the lowest average value of 45.36 ± 1.03. The smallest PDI obtained in emulsion prepared with 1: 1 FRR represented the most stable sample. A flow rate ratio of 3, yielded emulsions with the lowest stability after preparation, and following storage conditions result in a 43.7% increase in PDI. Although the average particle size of all prepared emulsions with different FRRs exhibited no significant changes during storage time, the PDI value of samples prepared with ≥ 2 FRRs, displayed robust fluctuations, indicating an instable emulsion (Fig. 5B). It is generally considered that the increase in the flow rate ratio leads to a decrease in droplet size, due to both a faster process of mixing and an increased dilution effect that consequently accelerates the diffusion between two organic and aqueous phases^25^. This is similar to when nanoparticles are produced by the precipitation method. When mixing time is shorter than the time needed for aggregation, the solution supersaturates, and nucleation events control the particle formation to produce smaller sizes after complete mixing is attained. In addition, when higher FFRs are applied, the final concentration of ethanol (organic phase) is reduced. This leads to a decrease in the production of larger droplets as a result of particle fusion, as well as lipid exchange (Ostwald ripening), when complete mixing is obtained^29^.

The droplet size and PDI of prepared emulsions, as a function of oil concentration (%), were also monitored during storage. Figure 5C shows that increasing the oil concentration from 0.167% to 0.25% led to significant increases in both droplet diameter (24.6%) and emulsion PDI (69.5%). This was followed by a decrease before reaching a plateau when 0.33–1.5% oil was used in the organic phase. The best result obtained was with the application of 0.5% oil concentration in organic phase. However, nanoemulsions prepared with 1.0% oil were consistently more stable during storage. Figure 5D shows the drop size and PDI dependence on surfactant concentration for drops created at 12 mL/min total flow rate and an FRR = 1.5. Emulsions with higher surfactant concentrations had larger droplet sizes and were more prone to aggregation. While emulsions containing 0.5% surfactant resulted in the smallest particles (61.91 ± 1.31 nm), the most stable emulsion was obtained by using a 2.5% surfactant concentration (PDI = 0.026). Generally, the formulations prepared with 1.0–5.0% surfactant exhibited more stability upon storage with less PDI variations and fluctuations. A previous study producing O/W emulsions using microfluidic chip technology monitored the stability of prepared emulsions^30^. These workers reported that the drop size was inversely dependent on surfactant concentration and tended to increase up to a critical concentration of micelle.

Surfactants have significant roles in creating a stable nanoemulsion by decreasing the interfacial tension that leads to reduced energy required to keep the two phases apart. Surfactants also inhibit the coalescence of new formed drops and facilitate the fusion which can exist between nanodroplets and the dispersed microscale droplets. The droplet size of an emulsion is decreased by increasing the surfactant concentration, due to higher interfacial area and lower interfacial tension^31^. In our study, increasing the surfactant concentration directly affected the emulsion droplet size, which did not follow the above-mentioned rule. In addition to its concentration, surfactants affect emulsion properties, such as particle size and PDI, depending on the critical micelle concentration (CMC). It has been shown that the addition of a surfactant at concentrations higher than its CMC did not guarantee the equilibrated surface tension needed in the microfluidic system, if the value of the CMC was small^32^. In our study, we used Tween 80 with a small CMC value (0.01)^33^, at a higher amount than Span 80, which may account for our observations reported herein. The surfactant concentration needed for achieving optimal results in our experiment was < 0.5%. Hence, further studies are required to examine the effect of different surfactant/oil ratios, surfactants with distinct CMC values and various process conditions, including total flow rate and flow rate ratio, on particle size of HSO emulsion.

### In-vitro release behaviour study

**Figure 6:**
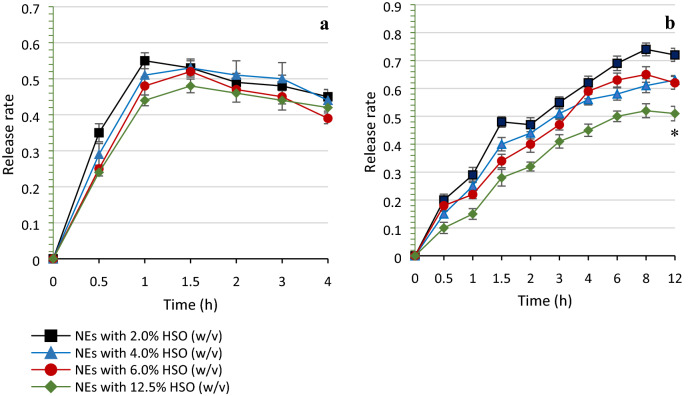
In-vitro release profile of linoleic acid from nanoemulsion with different concentrations of HSO as a function of incubation time in simulated gastrointestinal fluids (**a**: pH = 1.3, **b**: pH = 7.0). Each point represents the mean value ± SD (*n* = 3). *Significant values (*p* < 0.05) compared to other HSO concentrations (2, 4 and 6%).

An *in-vitro* release behaviour study of the HSO nanoemulsion was conducted to predict possible degradation of the nanoemulsion when exposed to gastrointestinal fluids. We were also interested in determining the initial concentration of HSO that would be optimal for process optimization. For this reason, and to evaluate the effect of oil concentration on release behaviour of nanoemulsion, we tested four concentrations (2, 4, 6 and 12.5%) (v/v) of our HSO emulsion systems prepared with a constant ratio of emulsifier/co-emulsifier (3:1). We compared emulsion stability in both simulated gastric fluid (SGF; pH = 1.3) and simulated intestinal fluid (SIF; pH = 7.0), during incubation times of 4 and 12 h, respectively. LA, the principle HSO fatty acid, was used as the chemical marker to evaluate release behavior. Figure 6a shows that all concentrations of HSO represented a similar release profile in SGF, characterized by a burst rate (0.44–0.55) that occurred in the initial 1 h; followed by a reduced LA release rate during the next 3 h of incubation. LA release rate from nanoemulsions was slowest when HSO was used at 12.5% (v/v).

During the first 3 h of incubation, the nanoemulsion containing 12.5% of HSO showed a slower release rate compared to other concentrations. It also displayed the best protective effects after 12 h of incubation at pH = 7.0 (Fig. 6b). This emulsion formulation had higher entrapment efficiencies, smaller particle size and dispersity (PDI) as well. The smaller sized nanoemulsion droplets typically result in greater release due to increased surface area of the particles. However, higher HSO emulsion entrapment, which was achieved in the 12.5% HSO nanoemulsion (> 99.0%) appeared to lower release rate. Furthermore, according to the Einstein-Stokes law for a diffusion-driven process, the prolonged release behaviour of lipid carriers can also be affected by the inverse of the lipid viscosity^34^. Hence, increasing the HSO concentration in the nanoemulsion formulation could also result in higher internal viscosity of lipid nanoparticle and slower release rates.

The zeta potential (ζ) refers to the electrophoretic movement of particles in an electric field medium, with a higher electric charge indicating stronger repulsive forces between particles that can hinder the aggregation and result in a more stable nanoemulsion^35^. Due to the unique emulsifying properties of HSO, the emulsion particles were abundantly more stable at higher concentrations of HSO.

### Effect of emulsification on Caco-2 epithelial transport of LA from HSO

**Figure 7:**
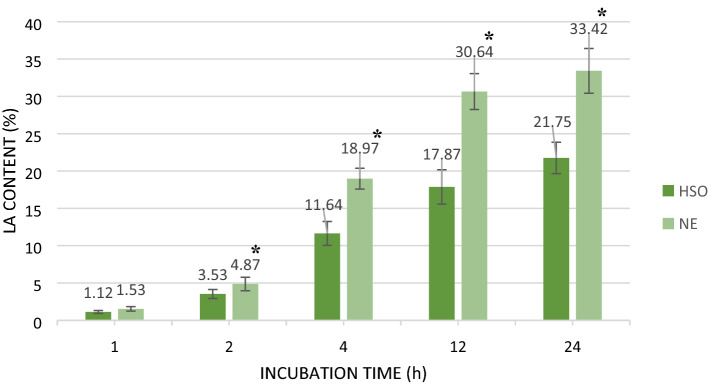
Linoleic acid (LA) recovery in basolateral chamber of Caco-2 cell culture treated with HSO and nanoemulsion. Each point represents the mean value ± SD (*n* = 3). *Significant values (*p* < 0.05) in comparison with HSO.

Caco-2 cells cultured to form a monolayer were used to measure intestinal epithelium LA transport derived from the nanoemulsion. The aim of this experiment was to compare the difference in LA transport across intestinal epithelium between the HSO (control sample) and the HSO formulated nanoemulsion over 24 h period. All emulsion samples representing optimized content (125 mg/mL of HSO) and with dilutions (2 times and 4 times) were previously tested for cell toxicity based on MTT redox assay. No toxic effect toward Caco-2 cell viability was observed with all samples (e.g. all cell viabilities were > 98% viable). In subsequent transport studies, results showed that during the first 2 h of incubation, the LA recovered in the basolateral chamber was negligible for both samples. However, starting from 4 h of incubation, LA concentrations were higher in the basolateral chamber when cells were treated with the nanoemulsion, compared to the control (*p* < 0.05). Extending the incubation further showed that LA derived from the HSO nanoemulsion formulation was transported 38.2% more, on average, than from the control sample. The transepithelial electric resistance (TEER) value was found greater than 300 Ω × cm^2^ for each sample tested, confirming the formation of monolayer atop the insert as depicted in Fig. 2. This confirms that the LA content recorded in the basolateral section of the well a result of active transport across the monolayer. Our findings confirm those of Heo et al. (2017), for conjugated linoleic acid (CLA) using a nanoemulsion containing 5% soy lecithin, 20% CLA and 65% of aqueous phase of glycerol solution on Caco-2 cells^36^. In their study, CLA uptake into triglyceride was significantly higher in nanoemulsion treated cells than the control. Since droplet size is an important factor affecting the lipase activity in the small intestine, nanoemulsions with smaller droplet size, and a larger surface area would be subjected to greater hydrolysis to enhance digestion and this would subsequently enable greater free fatty acid uptake, compared to a nanoemulsified lipid. This would explain the higher amounts of LA recovered in the basolateral chamber of Caco-2 cells treated with the HSO nanoemulsion, compared to cells treated with only the HSO (Fig. 7). This experiment would be the first step for future studies regarding determination of uptake and permeation rate of formulated HSO nanoemulsion across intestinal epithelium cells model.

## Conclusion

We studied the feasibility of two different approaches to prepare an HSO nanoemulsion for use in oral delivery purposes. Using lecithin/poloxamer 188 as the emulsifier/co-emulsifier, the optimal ultrasonic process condition for nanoemulsions was favourably suitable for developing an HSO-based nanoemulsion. The concentration of incorporated lecithin strongly affected the characteristics of the nanoemulsion including entrapment efficiency (%), particle size (nm), PDI, and zeta potential value. We confirmed the uniform structure of nanoemulsion by using cryo-TEM. The prepared formulation also displayed noted in-vitro release behaviour in simulated gastrointestinal fluids, specific to the gut and intestine, respectively. Hence, the formulated HSO nanoemulsion have an important role to promote sustained release of other poor aqueous-soluble drugs and improve bioavailability when used as a nanolipid carrier. Initial in-vitro tests using Caco-2 cell revealed that the nanoemulsions prepared in this study had the potential to enable greater transportation of LA in vitro, compared to the HSO itself. Further studies are needed to evaluate the permeation rate as well as confirm cellular uptake.

We also assessed HSO nanoemulsions prepared by microfluidics technology. This work has inferences for emulsion storage in droplet-based microfluidics and may have advantages for improved stability of nanoemulsion droplets. The size of the nanodroplets was expediently reduced to 61.91 and 44.74 nm by increasing total flow rate to 12 mL/min and aqueous: organic flow rate ratio to 4:1, respectively. It was concluded that the FRR parameter had the most pronounced effect on the droplet size and emulsion stability. While surfactant concentration was expected to decrease the droplet size by lowering the interfacial tension, the opposite effect was observed. Further work should focus on the optimization of surfactant/oil ratio and process conditions for nanoemulsions prepared by microfluidics technology. The stability and storage life results for this nanoemulsion system indicate a potential use of these formulations for drug delivery.

## Supplementary Information

Supplementary Information
