# Supplementary material for: Comparing microfluidics and ultrasonication as formulation methods for developing hempseed oil nanoemulsions for oral delivery applications
Source: Sci Rep. 2021 Jan 8;11:72. doi: 10.1038/s41598-020-79161-w (PMC7794282; doi:10.1038/s41598-020-79161-w)
Supplement: Supplementary file 1 — Supplementary Information [file 41598_2020_79161_MOESM1_ESM.docx]

**Supplement documents for the Manuscript:**

**Comparing Microfluidics and Ultrasonication as formulations method for developing hempseed oil nanoemulsions for oral delivery applications**

Farahnaz Fathordoobady^1^, Natalia Sannikova^2^, Yigong Guo^1^, Anika Singh^1^, David Kitts^1^, Anubhav Pratap-Singh^1^*

^1^ Food Nutrition and Health Program, Faculty of Land and Food Systems, The University of British Columbia,

2205 East Mall, Vancouver, BC V6T 1Z4

^2^ Ascension Sciences Inc., Vancouver, BC

*Correspondence: anubhav.singh@ubc.ca

**Supplement Table 1.** Experimental design and response variables for optimization of nanoemulsion

| Run  Order | Pt  Type | HSO Cons (%) | Poloxamer  Cons  (%) | Lecithin Cons  (%) | Time  (min) | Particle size  (nm) | PDI | Zeta potential | EE  (%) |
| --- | --- | --- | --- | --- | --- | --- | --- | --- | --- |
| 1 | 1 | 10 | 5 | 5 | 5 | 283.7 | 0.385 | 24 | 99.8 |
| 2 | 1 | 10 | 5 | 0 | 5 | 383.6 | 0.525 | 10.5 | 65.2 |
| 3 | 1 | 10 | 1 | 0 | 20 | 343.8 | 0.484 | 6.3 | 40.1 |
| 4 | 0 | 7.5 | 3 | 2.5 | 12.5 | 200.3 | 0.176 | 27.1 | 99.8 |
| 5 | 1 | 5 | 5 | 5 | 20 | 179.9 | 0.242 | 42.8 | 99.7 |
| 6 | 1 | 10 | 1 | 0 | 5 | 362.8 | 0.515 | 19.3 | 38.5 |
| 7 | 1 | 5 | 5 | 0 | 5 | 253.1 | 0.369 | 4.5 | 69.5 |
| 8 | 0 | 7.5 | 3 | 2.5 | 12.5 | 202.6 | 0.195 | 47.8 | 99.9 |
| 9 | 1 | 10 | 5 | 5 | 20 | 220.4 | 0.247 | 48.1 | 99.6 |
| 10 | 0 | 7.5 | 3 | 2.5 | 12.5 | 192.4 | 0.185 | 40 | 99.9 |
| 11 | 1 | 5 | 1 | 0 | 5 | 263.9 | 0.451 | 37 | 46.2 |
| 12 | 1 | 5 | 1 | 5 | 20 | 294.1 | 0.476 | 21.6 | 99.8 |
| 13 | 1 | 10 | 1 | 5 | 5 | 280 | 0.432 | 40.5 | 99.6 |
| 14 | 1 | 10 | 1 | 5 | 20 | 210.9 | 0.251 | 44.3 | 99.4 |
| 15 | 1 | 5 | 1 | 0 | 20 | 423.1 | 0.833 | 11.3 | 43.4 |
| 16 | 0 | 7.5 | 3 | 2.5 | 12.5 | 201.8 | 0.218 | 41.6 | 99.8 |
| 17 | 1 | 5 | 5 | 0 | 20 | 277.8 | 0.419 | 19.6 | 50.5 |
| 18 | 1 | 5 | 5 | 5 | 5 | 208.3 | 0.321 | 47.4 | 99.9 |
| 19 | 1 | 10 | 5 | 0 | 20 | 505.5 | 0.577 | 25.6 | 67.5 |
| 20 | 1 | 5 | 1 | 5 | 5 | 212.5 | 0.33 | 35.9 | 99.7 |
| 21 | 0 | 7.5 | 3 | 2.5 | 12.5 | 191.8 | 0.16 | 32.9 | 99.7 |
| 22 | -1 | 7.5 | 3 | 2.5 | 27.5 | 175.4 | 0.154 | 47.8 | 99.5 |
| 23 | 0 | 7.5 | 3 | 2.5 | 12.5 | 206.6 | 0.24 | 49.5 | 99.6 |
| 24 | -1 | 7.5 | -1 | 2.5 | 12.5 | 223.7 | 0.268 | 45.4 | 98.7 |
| 25 | -1 | 7.5 | 3 | -2.5 | 12.5 | 332.1 | 0.485 | 18.2 | 85.6 |
| 26 | -1 | 7.5 | 7 | 2.5 | 12.5 | 198.7 | 0.213 | 46.2 | 99.1 |
| 27 | -1 | 12.5 | 3 | 2.5 | 12.5 | 274.7 | 0.252 | 37.3 | 98.9 |
| 28 | -1 | 7.5 | 3 | 7.5 | 12.5 | 194 | 0.184 | 47.6 | 99.6 |
| 29 | -1 | 7.5 | 3 | 2.5 | -2.5 | 345.5 | 0.471 | 37.6 | 95.6 |
| 30 | -1 | 2.5 | 3 | 2.5 | 12.5 | 162.9 | 0.222 | 45.5 | 99.2 |

**Supplement Table 2.** Fatty acids identified by GC/MS in HSO^1^

| **Fatty acid** | **Retention time (min)** | ***m/z[M+H] ^+^** | ****Content (%)** |
| --- | --- | --- | --- |
| Palmitic acid (C16:0) | 9.18 | 256.4 | 5.16± 0.08 |
| Stearic acid (C18:0) | 10.40 | 284.48 | 2.53± 0.04 |
| Oleic acid (C18:1, ꞷ_9_) | 10.53 | 282.47 | 6.76± 0.20 |
| Linoleic acid (C18:2, ꞷ_6_) | 10.86 | 280.44 | 60.31± 0.86 |
| γ -linolenic acid (C18:3, ꞷ_6_) | 10.98 | 278.43 | 4.65± 0.08 |
| α-linolenic acid (C18:3, ꞷ_3_) | 11.24 | 278.43 | 18.94± 0.14 |
| Stearidonic acid (C18:4, ꞷ_3_) | 11.41 | 276.4 | 1.03± 0.03 |
| Eicosenoic acid (C20:1, ꞷ_9_) | 11.68 | 310.51 | 0.34± 0.04 |
| Decosanoic acid (C22:0) | 11.81 | 340.58 | 0.28± 0.01 |
| PUFA |  |  | 83.90 |
| MUFA |  |  | 7.10 |
| SFA/UFA |  |  | 0.09 |
| ꞷ_6_/ꞷ_3_ |  |  | 3.25/1 |

* GC-MS results are based on employing the *m*/*z* corresponding to the molecular ions [M + H] ^+^

** Values are the mean and standard deviation for three experimental determinations


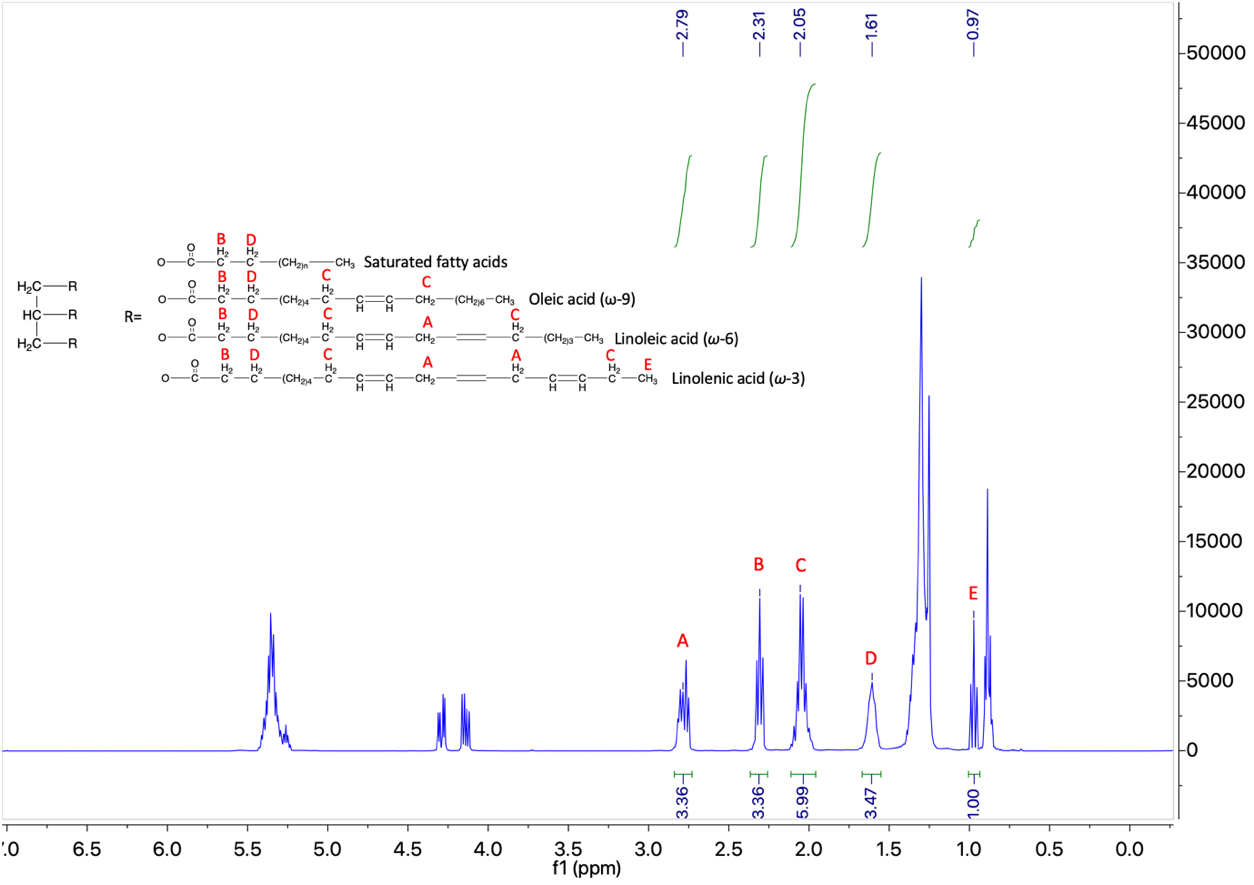


**Supplement Fig 1.** ^1^H NMR spectrum of hempseed oil (HSO) in CDCl_3_

**Supplement** **Fig. 2.** LC-MS results based on employing the *m*/*z* corresponding to the molecular ions [M + H] ^+^


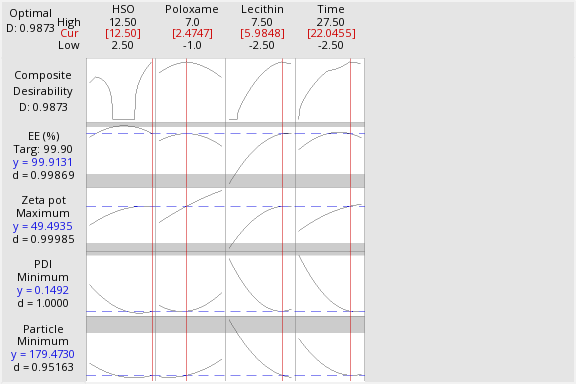


**Supplement Fig. 3** Graphical optimization of ultrasound process condition
